# Systemic Review: Is an Intradiscal Injection of Platelet-Rich Plasma for Lumbar Disc Degeneration Effective?

**DOI:** 10.7759/cureus.8831

**Published:** 2020-06-25

**Authors:** Takashi Hirase, Robert A Jack II, Kyle R Sochacki, Joshua D Harris, Bradley K Weiner

**Affiliations:** 1 Orthopedics and Sports Medicine, Houston Methodist Hospital, Houston, USA

**Keywords:** platelet-rich plasma, degenerative disc disease, intradiscal injection

## Abstract

Current studies evaluating the outcomes of intradiscal platelet-rich plasma (PRP) injections in degenerative disc disease (DDD) are limited. The purpose of this review was to determine if an intradiscal injection of PRP for degenerative discs results in a statistically significant improvement in clinical outcomes. A systematic review was performed using Preferred Reporting Items for Systematic Reviews and Meta-Analyses (PRISMA) guidelines. Level I-IV investigations of intradiscal PRP injections in DDD were sought in multiple databases. The Modified Coleman Methodology Score (MCMS) was used to analyze the methodological quality of the study. Only the outcome measurements used by more than 50% of the studies were included in the data analysis. The study heterogeneity and nature of evidence (mostly retrospective, non-comparative) precluded meta-analysis. Pre and post-injection pain visual analog scales (VAS) were compared using two sample Z-tests. Five articles (90 subjects, mean age 43.6 ± 7.7 years, mean follow-up 8.0 ± 3.6 months) were analyzed. Four articles were level IV evidence and one article was level II. Mean MCMS was 56.0 ± 10.3. There were 43 males and 37 females (10 unidentified). Pain VAS significantly improved following lumbar intradiscal PRP injection (69.7 mm to 43.3 mm; p<0.01). Two patients (2.2%) experienced lower extremity paresthesia after treatment. One patient (1.1%) underwent re-injection. No other complications were reported. In conclusion, intradiscal injection of PRP for degenerative discs resulted in statistically significant improvement in VAS with low re-injection and complication rates in this systematic review. It is unclear whether the improvements were clinically significant given the available evidence. The low level of evidence available (level IV) does not allow for valid conclusions regarding efficacy; however, the positive results suggest that further higher-quality studies might be of value.

## Introduction and background

Low back pain (LBP) is one of the most common causes of disability in the United States, with over 80% of American adults experiencing one or more lifetime episodes [1-2]. Although various organic and inorganic pathologies may cause LBP, degenerative disc disease (DDD) accounts for more than 40% of chronic LBP in the United States [3-4]. In spite of the high prevalence and morbidity associated with DDD, current treatment options are limited. Common treatments of early disease consist of a combination of conservative measures, such as bed rest, non-steroidal anti-inflammatory drugs (NSAIDs), physical therapy, and analgesic injections, which have shown to decrease symptoms but do not slow the progression of the disease [5-8]. Treatment of later disease consists of surgical approaches, including discectomy and spinal fusion, which are invasive, expensive, and have high rates of postoperative complications [9-13].

In recent years, platelet-rich plasma (PRP) has emerged as a relatively non-invasive treatment option for DDD unresponsive to conservative measures [14]. PRP is an autologous concentrate of various cells and growth factors acquired from centrifuged whole blood with growing evidence of its application in the healing response across different specialties, particularly in orthopedics. PRP has been shown to achieve its effects by delivering a high concentration of growth factors, including transforming growth factor-β, insulin-like growth factor, platelet-derived growth factor, and vascular endothelial growth factor, which activate cell proliferation and differentiation of vascularized cells [15]. Thus, various studies indicate its effective application in areas where vascularity is relatively preserved, including ligament, tendon, and muscle pathologies such as osteoarthritis, tendinopathies, lateral epicondylitis, and muscular injuries [16-17]. On the other hand, the vascular supply to the human intervertebral disc recedes completely during the developmental process, leaving virtually no direct blood supply to the disc in a healthy adult [18]. Therefore, it may be hypothesized that growth factors will have minimal effect on the degenerated discs. However, PRP has also demonstrated to have an anti-inflammatory effect by decreasing pro-inflammatory mediators at the injected site primarily by reducing the transactivation of the inflammatory regulator, nuclear factor-kappa B, and by inhibiting the inflammatory enzymes cyclooxygenase 2 and 4, metalloproteinases, and disintegrins [19-21]. This latter effect of PRP makes it a potential injectable option for the management of discogenic pain in DDD.

Current studies evaluating the outcomes of intradiscal PRP injections in DDD are mostly limited to small case reports and retrospective studies. Thus, the purpose of this investigation was to determine if the intradiscal injection of PRP for DDD results in statistically significant improvement in clinical outcomes with low re-injection and complication rates. The authors hypothesized that the procedure results in statistically significant improvement in pain VAS with low re-injection and complication rates.

## Review

Methods

**Figure 1:**
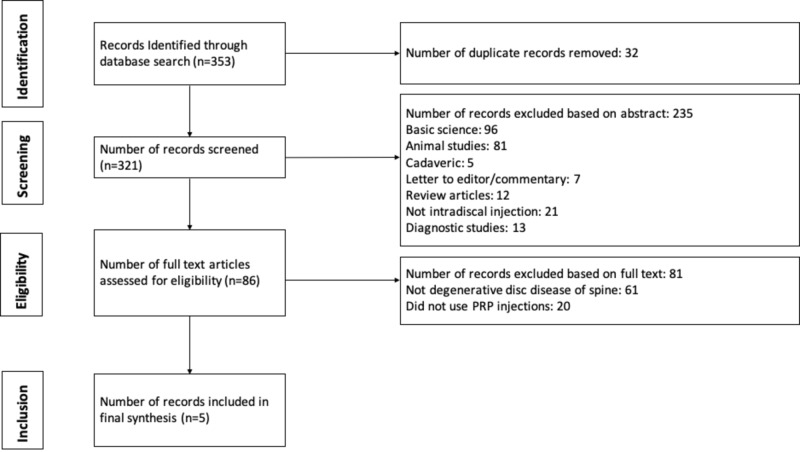
Flow diagram summarizing the literature search, screening, and review PRP (Platelet-Rich Plasma)

A systematic review was registered with PROSPERO (International Prospective Register of Systematic Reviews) on August 31, 2017 (Registration # CRD42017075843). PRISMA guidelines were followed [22]. Eligible studies consisted of level I-IV (via Oxford Centre for Evidence-Based Medicine (CEBM)) therapeutic studies that investigated the outcomes of intradiscal PRP injections for lumbar DDD among adult human patients [23]. The diagnosis was made in each included study based on a combination of history, physical examination, and radiographs, including magnetic resonance imaging (MRI) for every patient. Studies that included non-DDD etiology of back pain were excluded. Cadaveric studies, basic science and animal studies, diagnostic studies, economic studies, prognostic studies, level V evidence expert opinions, letters to editors, and review articles were excluded. Studies published in non-English languages were not excluded but were unidentified in the medical databases. In the event of different studies with duplicate subject populations, the study with the longer follow-up, higher level of evidence, greater number of subjects, or greater clarity of methods and results was included. The authors conducted separate searches of the following medical databases: MEDLINE, Web of Science, and Cochrane Central Register of Controlled Trials databases. Under the PROSPERO registration, similar prior systematic reviews and meta-analyses were sought and none were identified. The searches were performed on April 20, 2020. The search terms used were “platelet-rich plasma,” “degenerative disc,” “spine,” and “injection.” The search results were reviewed for duplicates and the inclusion criteria to determine articles that were included in the final analysis (Figure 1).

Two authors independently reviewed all articles using the methodology recommended by Harris et al. [24]. The study design, patient populations, and procedure technique were first identified. All lower back-specific patient-reported outcome scores, re-injection rates, and complication rates were analyzed.

The levels of evidence were then assigned based on the Oxford Centre for Evidence-Based Medicine [23]. Study methodological quality was analyzed using the Modified Coleman Methodology Score (MCMS) [25]. The overall Strength-of-Recommendation Taxonomy (SORT) score was B and Grading of Recommendations Assessment, Development, and Evaluation (GRADE) score was C [26-27]. Study heterogeneity and nature of evidence (mostly retrospective, non-comparative) precluded meta-analysis. Thus, a best-evidence synthesis was used instead [28]. Only the outcome measurements used by more than 50% of the studies were included in the data synthesis to increase the power of the measurement over that of individual studies. A weighted mean of pre- and post-injection values from each study was calculated and comparisons were made using two sample Z-tests (http://in-silico.net/tools/statistics/ztest) using a p-value of less than 0.05 for significance. The individual changes in LBP visual analog scale (VAS) were compared with a previously reported minimal clinically important difference (MCID) of 22.5, substantial clinical benefit (SCB) of 32.5, and a patient acceptable symptomatic state (PASS) of 33.5 [29-30].

Results

Five articles were analyzed (Table 1) [31-35]. Four articles were level IV evidence and one article was level II. According to MCMS, one article was good (scores between 70 to 84), three articles were fair (scores between 55 to 69), and one article was poor (scores less than 55). The mean MCMS was 56.0 ± 10.3. There were 90 patients analyzed. There were 43 males and 37 females (10 unidentified). The mean age was 43.6 ± 7.7 years old with a mean follow-up of 8.0 ± 3.6 months.

**Table 1:** Study demographics NR (Not Recorded); PRP (Platelet-Rich Plasma); IVD (Intervertebral Discs); FRI (Functional Rating Index); SF-36 (36-item Short-Form Health Survey); VAS (Visual Analog Score); PPI (Present Pain Intensity); ODI (Oswestry Disability Index); RDQ (Roland-Morris Disability Questionnaire); MODQ (Modified Oswestry Disability Questionnaire); SVF: Stromal Vascular Fraction; RDQ: Roland-Morris Disability Questionnaire

Study	Tuakli et al. 2016 [31]	Comella et al. 2017 [32]	Akeda et al. 2017 [33]	Levi et al. 2016 [34]	Bhatia et al. 2016 [35]
Type of study	Prospective, double-blind randomized controlled study	Case Series	Case Series	Case Series	Case Series
Level of evidence	II	IV	IV	IV	IV
No. subjects	29	15	14	22	10
Gender (M/F)	14/15	11/4	8/6	10/12	NR
Age (mean, years)	41.4	51.5	33.8	47.5	NR
Injection method	Fluoroscopy-guided single injection of autologous PRP into one or more symptomatic degenerative IVD	Fluoroscopy-guided injection of 1 ml of SVF/PRP suspension into one or more symptomatic IVD	Fluoroscopy-guided single injection of autologous PRP into one or more symptomatic degenerative IVD	Fluoroscopy-guided single injection of autologous PRP into one or more symptomatic degenerative IVD	Single injection of 5 mL autologous PRP into epidural space via interlaminar approach w/ 18 G needle
Follow-up (months)	12	6	10	6	3
Outcomes	VAS, FRI, SF-36 pain, SF-36 physical function	VAS, PPI, ODI	VAS, RDQ	VAS, ODI	VAS, MODQ
Post-injection treatments	No	No	No	No	No
Post-injection cryotherapy	No	No	No	No	No
Use of NSAIDs (few days pre-injection and immediate post-injection)	No	No	Yes – post-injection for unbearable pain	No	No

PRP was obtained in all studies by the centrifugation of 30 to 200 mL of autologous blood to perform a fluoroscopy-guided injection of 1 to 5 mL of PRP directly into one or more symptomatic lumbar intervertebral discs (Table 2). Three studies confirmed symptomatic discs using provocative discography. The remaining two studies utilized MRI alone with a combination of history and physical exam. All studies performed intradiscal PRP injections after the failure of non-interventional management. None of the studies recorded the use of post-injection cryotherapy. One study approved the use of post-injection NSAIDs for unbearable pain. No study compared leukocyte-poor PRP to leukocyte rich PRP. However, one study reported using leukocyte-poor PRP, and one study reported using leukocyte-rich PRP. One study used a negative control with a placebo contrast injection and reported a significant improvement in FRI as compared to controls at eight weeks post-treatment but reported no significant difference in VAS and SF-36 pain at any time post-treatment. No comparison injections were made in all other studies.

**Table 2:** PRP preparation NR (Not Recorded); PAW classification (classification system for PRP that incorporates platelet concentration, activation method, and white blood cell count); PRP: Platelet-Rich Plasma

Study	Tuakli et al. 2016 [31]	Comella et al. 2017 [32]	Akeda et al. 2017 [33]	Levi et al. 2016 [34]	Bhatia et al. 2016 [35]
PRP Spinning Approach	NR	Single	Double	Single	NR
Duration of Spin (Minutes)	NR	8	15 and 15	14	NR
Company	Harvest Technologies Corporation, Plymouth, MA, USA	NR	Kawasumi Laboratories, Inc., Tokyo, Japan	Harvest Technologies Corporation, Plymouth, MA, USA	NR
PRP Activator	NR	NR	CaCl_2_	NR	NR
PRP Volume Injected (ml)	1-2	1	2	3	5
Platelet Concentration	NR	NR	3.7 x baseline	NR	NR
White Blood Cell Count	NR	NR	1/120 of baseline	High	NR
PAW Classification	NR	NR	P3-B	NR	NR

VAS decreased by 26.4 mm at six months following intradiscal PRP injection (Table 3; p<0.01). However, only two studies (32 patients) reported individual data to allow a direct comparison of the change in VAS with previously reported MCID, SCB, and PASS. Of the two studies, only 19 patients (59.4%) met MCID and 12 patients (37.5%) met SCB and PASS.

**Table 3:** Individual Study Outcome Measures NR (Not Recorded); PRP (Platelet-Rich Plasma); F/U (Follow-Up); FRI (Functional Rating Index); SF-36 (36-item Short-Form Health Survey); VAS (Visual Analog Score); PPI (Present Pain Intensity); ODI (Oswestry Disability Index); RDQ (Roland-Morris Disability Questionnaire); MODQ (Modified Oswestry Disability Questionnaire); LE (Lower Extremity)

Study		Tuakli et al. 2016 [31]	Comella et al. 2017 [32]	Akeda et al. 2017 [33]	Levi et al. 2016 [34]	Bhatia et al. 2016 [35]
FRI	Baseline	51.47 + 15.62	NR	NR	NR	NR
	Final F/U	33.98 + 20.35	NR	NR	NR	NR
SF-36 Pain	Baseline	43.28 + 21.11	NR	NR	NR	NR
	Final F/U	67.79 + 23.51	NR	NR	NR	NR
SF-36 Physical Function	Baseline	56.40 + 18.52	NR	NR	NR	NR
	Final F/U	73.20 + 19.38	NR	NR	NR	NR
VAS	Baseline	79.8 + 15.6	56	75 + 13	66.0 + 12.2	61.0 + 12.0
	Final F/U	58.2 + 23.3	36	29 + 28	41.4 + 27.0	37.0 + 6.7
PPI	Baseline	NR	2.6	NR	NR	NR
	Final F/U	NR	1.8	NR	NR	NR
ODI	Baseline	NR	NR	NR	31.0 + 9.8	NR
	Final F/U	NR	NR	NR	23.5 + 16.2	NR
RDQ	Baseline	NR	NR	12.6 + 4.1	NR	NR
	Final F/U	NR	NR	2.8 + 3.9	NR	NR
MODQ	Baseline	NR	NR	NR	NR	49.2 + 9.6
	Final F/U	NR	NR	NR	NR	29.5 + 11.6
Complications		0	0	2 – LE paresthesias	0	0
Re-Injection		0	0	0	1	0

Re-injection and complication rates were minimal. There was one patient (1.1%) that required re-injection (Table 4). There were two cases (2.2%) of transient lower extremity paresthesia in unspecified nerve distributions that occurred one and six months post-treatment, both of which self-resolved within seven days. No other complications were reported.

**Table 4:** Average study outcome measures included in best-evidence synthesis VAS (Visual Analog Score); F/U (Follow-Up)

	VAS
Baseline	69.7 + 13.6
Final F/U	43.3 + 23.5
p-value	<0.001

Discussion

It was determined that an intradiscal injection of PRP for DDD results in a statistically significant improvement in VAS. Although all reviewed studies presented statistical significance in the improvement of VAS after an intradiscal PRP injection for DDD (69.7 mm to 43.3 mm; p<0.01), no studies analyzed the clinical importance of outcome scores. Various studies have shown that a statistically significant score change in outcomes does not imply a clinically significant change [36-40]. Thus, measuring the MCID was introduced to determine the smallest difference in outcome score that patients found beneficial [41]. SCB is comparable to MCID but seeks to further develop a standard that better reflects the envisioned benefit of an intervention [42]. PASS is also a similar concept but instead represents the maximum amount of signs and symptoms beyond which patients consider themselves well [43]. Park et al. determined in a study of 105 patients with persistent LBP after lumbar surgery that the MCID and SCB for LBP VAS are 22.5 mm and 32.5 mm, respectively [29]. Furthermore, Tuback et al. determined in a study of 330 ankylosing spondylitis patients that the PASS of LBP VAS is 33.5 mm [30]. Of the two reviewed studies that reported individual data, only 19 patients (59.4%) met MCID and 12 patients (37.5%) met SCB and PASS. This demonstrates that though the procedure results in statistically significant improvement, a large portion of the patients does not achieve a clinically meaningful improvement in outcomes.

All studies analyzed utilized an intradiscal injection of PRP to treat both the symptoms and the progression of disc degeneration in DDD. One of the analyzed studies by Comella et al. also utilized stromal vascular fraction (SVF) obtained from a mini-lipoaspirate procedure of fat tissue to be injected into the disc as a PRP-SVF suspension [32]. The authors hypothesized that SVF, which is a mixture of growth factors and adipose-derived stem cells (ADSCs), can be injected into the disc simultaneously with PRP to minimize inflammation while promoting healing. This study of 15 patients resulted in an average VAS decrease of 20.0 mm at the six-month follow-up with no adverse effects, which was neither superior nor inferior to other studies that utilized PRP alone.

Numerous types of PRP systems exist, with varying leukocyte, platelet, and growth factor concentrations. Leukocytes consist of neutrophils, eosinophils, basophils, lymphocytes, and monocytes, which are responsible for providing an acute and chronic inflammatory response against foreign invaders. Studies comparing leukocyte-rich and leukocyte-poor PRP have demonstrated a significantly higher inflammatory response and cell death seen with leukocyte-rich PRP [44-45]. Of the studies included in the review, Levi et al. used leukocyte-rich PRP, which showed a significant decrease in VAS (24.6 mm) at the six-month follow-up [34]. Akeda et al. used leukocyte-poor PRP and reported a larger decrease in VAS (43.0 mm) at the six-month follow-up [33]. However, this review was unable to develop conclusions regarding outcome differences in the use of leukocyte-rich versus leukocyte-poor PRP, as none of the reviewed studies directly compared the use of these formulations.

Complication and re-injection rates after intradiscal PRP injection were low. The re-injection rate in this study was 1.1%. Furthermore, besides the 2.2% incidence of transient paresthesia post-injection, there were no reported adverse effects compared to the higher rates of complications with surgery such as infection, hematomas, thromboembolic events, and adjacent level disease. Overall, this study demonstrates that intradiscal injection of PRP for DDD leads to clinical improvement with low complication and re-injection rates. However, further higher-quality studies with randomized controlled trials are necessary to justify the use of PRP over more cost-effective treatment methods.

There are several limitations among the studies included in this review. Four of the five articles were level IV evidence, which limits the strength of the results [31-34]. None of the studies used a double-blinded approach producing potential bias. The average study methodological quality as assessed by the MCMS was fair. The assimilation of heterogeneous, low methodological-quality studies with VAS is a significant limitation. However, the authors minimized this as much as possible with strict study eligibility and inclusion criteria, despite the level IV evidence nature of the studies. Furthermore, the heterogeneity of outcome measures used among the studies limited the data analysis to one outcome measure. Additionally, MCID, SCB, and PASS are used to compare individual differences between preoperative and postoperative outcomes, and a majority of the reviewed studies reported means of patients and did not include individual statistics. Future studies can improve through designing a prospective comparative trial, increasing study size, and standardizing clinical outcome measures such as using VAS, ODI, numeric rating scale (NRS), and functional rating index (FRI) simultaneously. Another possible limitation of this review is that other relevant studies on this topic could have been excluded, despite conducting a systematic search.

## Conclusions

Intradiscal injection of PRP for degenerative disc disease results in a statistically significant improvement in VAS with low re-injection and complication rates. Further randomized controlled studies that show a clinically relevant improvement in multiple outcome parameters are necessary to evaluate the true efficacy of this treatment.
